# Assessing physical access to healthy food across United Kingdom: A systematic review of measures and findings

**DOI:** 10.1002/osp4.563

**Published:** 2021-09-15

**Authors:** Elzbieta Titis, Rob Procter, Lukasz Walasek

**Affiliations:** ^1^ Department of Computer Science Warwick Institute for the Science of Cities University of Warwick Coventry UK; ^2^ Department of Computer Science University of Warwick Coventry UK; ^3^ Alan Turing Institute London UK; ^4^ Department of Psychology University of Warwick Coventry UK

**Keywords:** food access, food desert, obesity, RFE

## Abstract

**Background:**

Existing research suggests that physical access to food can affect diet quality and thus obesity rates. When defining retail food environment (RFE) quantitatively, there is a little agreement on how to measure “lack of healthy food” and what parameters to use, resulting in a heterogeneity of study designs and outcome measures. In turn, this leads to a conflicting evidence base being one of the many barriers to using evidence in policy‐making.

**Aims:**

This systematic review aimed to identify and describe methods used to assess food accessibility in the United Kingdom (UK) to overcome heterogeneity by providing a classification of measures.

**Materials & Methods:**

The literature search included electronic and manual searches of peer‐reviewed literature and was restricted to studies published in English between January 2010 and March 2021. A total of 9365 articles were assessed for eligibility, of which 44 articles were included in the review. All included studies were analysed with regards to their main characteristics (e.g., associations between variables of interest, setting, sample, design, etc.) and definition of RFE and its metrics. When defining these metrics, the present review distinguishes between a point of origin (centroid, address) from which distance was calculated, summary statistic of accessibility (proximity, buffer, Kernel), and definition of distance (Euclidean, network distance). Trends, gaps and limitations are identified and recommendations made for food accessibility research in UK.

**Results:**

Multiple theoretical and methodological constructs are currently used, mostly quantifying distance by means of Euclidean and ring‐buffer distance, using both proximity‐ and density‐based approaches, and ranging from absolute to relative measures. The association between RFE and diet and health in rural areas, as well as a spatiotemporal domain of food access, remains largely unaccounted.

**Discussion:**

Evidence suggests that the duration of exposure may bear a greater importance than the level of exposure and that density‐based measures may better capture RFE when compared with proximity‐based measures, however, using more complex measures not necessarily produce better results. To move the field forward, studies have called for a greater focus on causality, individual access and the use of various measures, neighbourhood definitions and potential confounders to capture different aspects and dimensions of the RFE, which requires using univariate measures of accessibility and considering the overall context in terms of varying types of neighbourhoods.

**Conclusion:**

In order to render ongoing heterogeneity in measuring RFE, researchers should prioritise measures that may provide a more accurate and realistic account of people’s lives and follow an intuitive approach based on convergence of results until consensus could be reached on using some useful standards.

## 1 INTRODUCTION

Growing evidence shows that various properties of the retail food environment (RFE), sometimes referred to as the community nutrition environment, play an important role in influencing dietary health outcomes of individuals by making “unhealthy” foods more accessible and/or restricting access to “healthy” foods; these properties include number, type, location, and accessibility of outlets, in contrast to availability, price, and quality constituting the consumer nutrition environment.^1^ In addition, RFE may also influence diet‐related behaviors through such mechanism as driving desire for foods, normalizing behaviors, or establishing habits.^2^ Indeed, there is a strong link between availability of unhealthy food and diet‐related ill health, including obesity,^3^, ^4^, ^5^, ^6^ diabetes,^7^ and colorectal cancer.^8^ Specifically, when considering links with body mass index (BMI), various aspects of RFE have been studied, including (a) the double burden of fast food (FF) outlet exposure and low income in terms of their joint contribution to social inequalities in health^3^; (b) the relative density of unhealthy food retail outlets (FROs) around homes, schools, and routes to school showing positive associations with higher BMI in children^6^; and (c) and longitudinal pathways from multiple types of FROs to BMI through dietary behaviors, with results showing a positive association between higher numbers of FF outlets and lower numbers of sit‐down restaurants in neighborhoods and higher consumption of an obesogenic diet.^4^ Still, since other studies found negative or null associations between FF access and diet or BMI,^9^, ^10^ the evidence base remains inconclusive.

Research on RFE has also examined food access as a function of neighborhood racial and socioeconomic demographics, including distribution of food resources across the urban spectrum (nonurban, low‐density urban, and high‐density)^11^ and effects on environmental justice,^12^ with racial considerations being particularly strong in the US literature. For example, one study has found that African Americans in New York had fewer opportunities to obtain healthy foods and greater access to FF restaurants when compared to other ethnicities,^13^ whereas another study has reported predominantly Hispanic neighborhoods to have a higher proportion of FF restaurants than racially mixed neighborhoods.^14^ Evidence also suggests that the socioeconomic status of the neighborhood may affect both the presence of certain FROs and food quality.^15^ Others have studied RFE in combination with other environmental aspects of the neighborhood urban design, including physical activity (PA)‐related aspects^16^, ^17^ and transportation mode,^17^, ^18^ the latter including motor vehicle ownership^18^, ^19^ and public transport.^18^, ^20^, ^21^ Finally, a growing number of studies have examined specific temporality of FROs (e.g., opening and closing times) in terms of its alignment with individual's discretionary time for food shopping and before‐and‐after assessment of supermarket intervention on diet^22^, ^23^; for example, space–time accessibility measures have been used to study the availability of FROs in activity spaces accounting for individuals' differences and travel behaviors.^24^, ^25^

In the growing literature on RFE, researchers have used various datasets and analysis techniques to estimate regional variability in the barriers for accessing healthier food options and achieving more balanced diets. This variability may be responsible for the often conflicting results reported in these studies. These inconsistencies are problematic for the policy makers who wish to use this knowledge to inform their decision of how to best tackle the obesity epidemic. The purpose of the present review is to evaluate and classify these methods, and to identify knowledge gaps in the context of recent research on RFE, diet, and health outcomes. The focus on the present review is on the studies of RFEs in the United Kingdom. There are three reasons for this. First, this work has already been done in the United States; second, datasets available in the United Kingdom as well as obesogenic environment are very unique when compared with the United States and other countries; third, unique policy context in the United Kingdom requires addressing local relevance to improve using evidence in policy and practice.

Location‐based access to FROs has traditionally been measured using a proximity (proximity to the nearest store) or a buffer approach (e.g., presence/absence or number of food stores within an area). The former method relies on the distance to FROs measured in units of distance or travel time, whereas the latter quantifies the availability of FROs using kernel density estimation or spatial clustering, providing detail on the number of stores within a predefined distance. Recently, some metrics combined the two (distance and density), making use of Kernel density estimation to create estimates across a continuous surface, so that density can be calculated from any location, while considering the number of stores nearby with a weighting function, so that closer amenities are weighted more heavily than those located further away.^26^

Early attempts to map RFE used regional aggregates of simple distance metrics, such as straight line (Euclidean) distance, aka a ring‐buffer approach, measuring distance from an arbitrary focal point, for example, a geographical or population weighted centroid. With the advancement of techniques facilitating much narrowed geographical focus, the fine detail of a neighborhood could be captured using a network distance, accounting for road network distance, and travel time.^27^ Still, due to its simplicity, Euclidean distance remains the preferred distance for calculating Kernel estimates, meaning that possible barriers to travel are often disregarded.^26^ Recent trends in studying food access using sophisticated indices have shifted toward recognizing urban environments as dynamic systems, accounting for “travel burden,”^28^ wider mobility patters of consumers, activity spaces, and spatiotemporal determinants of access (e.g., shops' opening times).

To model RFE accurately, the environment needs to be defined for an actual shopping behavior of individuals. This definition, however, differs between individuals as given by their understanding of locality and appropriates of food access.^29^ Therefore, Chaix et al.^30^ have called on future studies to reject a uniform definition of shopping neighborhood in favor of approaches that will allow for applying individual‐specific scales based on socioeconomic differences. For the UK context, 1‐mile circular buffers centered on home and work addresses has been suggested as adults' definition of their local shopping neighborhood^31^; nevertheless, the neighborhood definition that is congruent with actual food‐shopping behaviors in terms of where individuals decide to shop for daily groceries remains unknown.^32^

In the growing literature on RFE, numerous measures of food access and their classifications have been proposed. Jaskiewicz et al.^33^ distinguish the following three categories of food access measures: (a) cumulative opportunity measures, including container (the number of stores within a given geographic area), coverage (the number of stores within a given distance), minimum distance (distance to the nearest store), and average distance to all stores in a geographic area; (b) gravity measures, which take into account the “cost” of distance and travel time, and include gravity kernel and two‐step floating catchment area; and (c) utility theory measures—computationally intensive and requiring detailed travel data. Feng et al.^34^ give examples of complexities of build environment metrics, and in addition to spatial access, consider such factors as density in terms of “the amount of activity found in an area,”^35^ diversity referring to “the spatial arrangement of land use” and influencing the distance and mode of travel, connectivity that allows people to connect with places and consequently affects experience of travelling, street design that is likely relevant to active transport, and composite indices that reflect interconnected nature of build environment metrics. In this review, we propose our own classification of food access measures based on the recent state of the art in the UK research.

Most food access measures account for accessibility and affordability of food retailers (e.g., availability of healthy foods, and food prices), and combine these characteristics with certain population characteristics, such as access to transportation and socioeconomic resources of food buyers, to estimate access at the individual level (e.g., when taking into account vehicle availability, the time cost of access to food, or perception of food access limitations), or for areas when the access of a neighborhood or specific geographic boundary is considered.^36^ Food deserts (FDs) and food swamps (FSs) are area‐based measures that remain important terms in the discussion of RFE. In the UK context, FD has been defined as a region further than 500‐m walking distance away from the closest supermarket selling a wide variety of healthful (fruits and vegetables) and relatively inexpensive foods^37^ that residents are limited in terms of economic resources (low‐income earners), mobility, and access to transport.^38^ In the United Kingdom, there is an ongoing debate questioning the very existence of FDs,^39^ and among those who agree on their existence, questions have been raised whether their presence affects food choices and obesity rates,^37^, ^40^ with a recent systematic review arguing that food access is socioeconomically patterned, thus rendering geographical access irrelevant.^41^

In the United States, research has moved from focusing on FDs to investigating FSs—underserved areas where calorie‐rich food is readily available at convenient stores and takeaway restaurant, but healthy food options are nonexistent or scarce—as recent evidence suggests FF outlets play a significant role in the nutrition, transforming communities into obesogenic environments. The research suggests that FSs are more prevalent than supermarkets and their presence can predict obesity rates better than FDs,^42^ in addition to being associated with greater rates of hospitalizations for complications among diabetic adults^43^; outside the United States., one Canadian study further points toward greater availability of FF outlets,^44^ whereas in the United Kingdom, another study found a negative association between obesity and FF outlet density in the South Asian group.^45^ US evidence further suggests that low‐income and racial‐ethnic minorities are more likely to live in FSs,^46^, ^47^ and that the race and ethnicity of a community shapes RFE, as FF outlets are more prevalent in ethnic minority areas.^9^ Drawing on these results, recent US policy has focused on reducing FDs by limiting FF outlets and small food stores while increasing access to supermarkets in low‐income neighborhoods, by introducing zoning restrictions on FF restaurants within 3 km of low‐income residents that could lower obesity rates by about 3%.^10^ As with the gaps in FDs research, research on FSs struggles to operationalize the concept for empirical analysis and to account for people self‐selecting into neighborhoods based on individual food preferences.^42^ In the United Kingdom, RFE has been found to encourage unhealthy foods consumption in poorest areas in which children are disproportionately exposed to both takeaways and more visible advertising for unhealthy foods^48^; research into the relationship between RFE, health, and diet is still underdeveloped and the evidence remains inconclusive.^49^

Despite the fact that the study of FDs originated in the United Kingdom, it is US research that has been pioneering the field in terms of creating considerable policy and academic attention leading to increased variability in measures.^50^ The former can be attributed to putting access to healthy food on government's equity agenda to address high levels of segregation, including ethnic, racial, and socioeconomic segregation,^51^ triggering issues of economic inequality and the systemic racism permeating America's food system. In addition, government statistics at small geographical levels are widely available online, advancing the field in terms of methods that can be used to study RFE. On the other hand, data scarcity in the United Kingdom restricts the use of more powerful analytical methods and food access has been mainly discussed in the context of food poverty.

Recent evidence suggests that 10.2 million individuals in Great Britain live in FDs that have been attributed to economic drivers of poor diet, such as food affordability and food prices including regional variations.^52^ Recently, Covid‐19 introduced new drivers of food insecurity, in addition to financial hardship faced by low‐income households, by limiting access to food in terms of basic supplies and through isolation.^53^ As a result, a newly vulnerable group who were financially stable pre‐Covid emerged, making reliance on overstretched food banks and food aid charities no longer a sustainable solution to food insecurity.^53^ Despite significant changes in behavior, national lockdown restrictions requiring closure of all restaurants except takeaway and delivery services had no overall adverse effect on obesity levels.^54^ This would be in line with qualitative research showing many customers experienced positive changes to household food behaviors, including increased home‐cooking and food sharing and increased attention to diet, likely encouraged by the restrictions and public health advice, such as stay at home.^55^ Nevertheless, the pandemic may have affected weight management in social lockdown, putting at increased risk people living with obesity and mental health problems.^56^

When considering the aforementioned perspectives on FDs and the country‐specific segregation levels and data conditioning, we argue that United Kingdom is a very different case study to the United States, where data availability allows for growing arsenal of measures, segregation levels are far less moderate^51^ and FDs are inextricably linked to racial inequality.^57^, ^58^ Moreover, European countries, including pre‐Brexit UK, have a stronger welfare and a different labor market, resulting in urban marginality having different forms than in the US context.^51^ Having focused specifically on the United Kingdom, this review addresses the needs of policy‐makers who require context‐specific evidence to ensure local relevance. Moreover, prior literature reviews reveal a methodologically heterogeneous but limited evidence base in regards to how the built environment should be measured and modeled.^34^ This may be compounded by a focus on more densely populated urban areas,^59^ neglecting smaller towns and villages. Identifying research trends and knowledge gaps will lead to the improved use of evidence in policy‐making relevant to the United Kingdom.

Moreover, these observations could be of value to other developed countries and regions having similar data scarcity issues and socioeconomic trends, as well as they could provide useful insights for `modeling obesity rates in other European countries with underdeveloped retail business that, however, follow in United Kingdom's footsteps in terms of undergoing changes in food systems and nutrition. Since investigations for continental Europe are scarce,^60^, ^61^, ^62^ this review is focused specifically on the United Kingdom to complement the evidence base being made of mostly United States and Canada studies that provide majority of evidence; for this reason, as well as to promote multilateral research and innovation initiatives, US studies are briefly addressed, including research on FSs. Africa, Australia, and Asian countries were excluded from the analysis as being outliers in terms of accelerated urbanization (Africa), regional isolation and produce seasonality (Australia), and rising population densities and consumption (Asia). Therefore, the primary aim of this systematic review is threefold: (a) to identify, critically evaluate, and classify the key measures that have been used to assess disparities in food access in United Kingdom over the last decade to provide the recent state of the art for classifying given regions as having inadequate access to healthy food; (b) to identify gaps and limitations to provide an evidence‐base for drawing recommendations for future UK research and policy; and (c) to compare the recent state‐of‐the‐art in UK studies with US research pioneering the field to offer additional insights for development and promote multilateral research and innovation initiatives subjected to data availability.

## 2 METHOD

The literature search included electronic and manual searches of peer‐reviewed literature in English published between January 2010 and March 2021. To identify relevant articles for this review, a search was made using the combination of general and specific food environment keywords (e.g., food OR nutrition OR diet AND community OR neighbourhood OR supermarket OR fast‐food, etc.) followed by measure‐related terms (assess* OR measure* OR metric* OR instrument*) and numerous additional terms corresponding to various levels of geography, including country (United Kingdom OR Britain OR England, etc.), region (“North East” or “North West,” etc.) and city, as well as geography‐related adjectives (British OR "Northern Irish" OR Scottish OR Welsh) and exclusions (England AND NOT “New England”); in addition to excluding continents and US states, the search included limiters for database specific remote categories. Three databases were searched for articles published between January 2010 and March 2020—PubMed, Scorpus, and Web of Science—followed by a review of the references of the articles identified from these databases (both primary studies and reviews). Only peer‐reviewed articles published in English were included. A detailed search strategy is provided in supporting information S1.

Articles were assessed in relation to (a) main characteristic of studies, including background (associations), setting (country), sample (e.g., adults, children), food store type (healthy vs. unhealthy), food environment (e.g., residential, school), neighborhood definition as given by various aspects of food scape metrics, for example, buffer size and distance type (800 m pedestrian road network buffers), research method (e.g., regression, spatial maps), unit of analysis (e.g., neighborhood), findings, conclusions, and study design (cross‐sectional vs. longitudinal); and (b) food scape metrics broken down into the three major aspects, including starting point (centroid, address), approach (proximity, buffer, Kernel), and distance (Euclidean, network).

### 2.1 Article screening

The first author (ET) developed the search strategy and conducted the database searches, identifying and collating all potentially relevant articles. The first author then screened all titles and abstracts of identified articles against the inclusion and exclusion criteria. Full texts of potentially eligible studies were then retrieved. When there was uncertainty regarding inclusion/exclusion of a specific paper, the other authors were consulted (RP and LW) until unanimous agreement was reached.

### 2.2 Data extraction and synthesis

**FIGURE 1:**
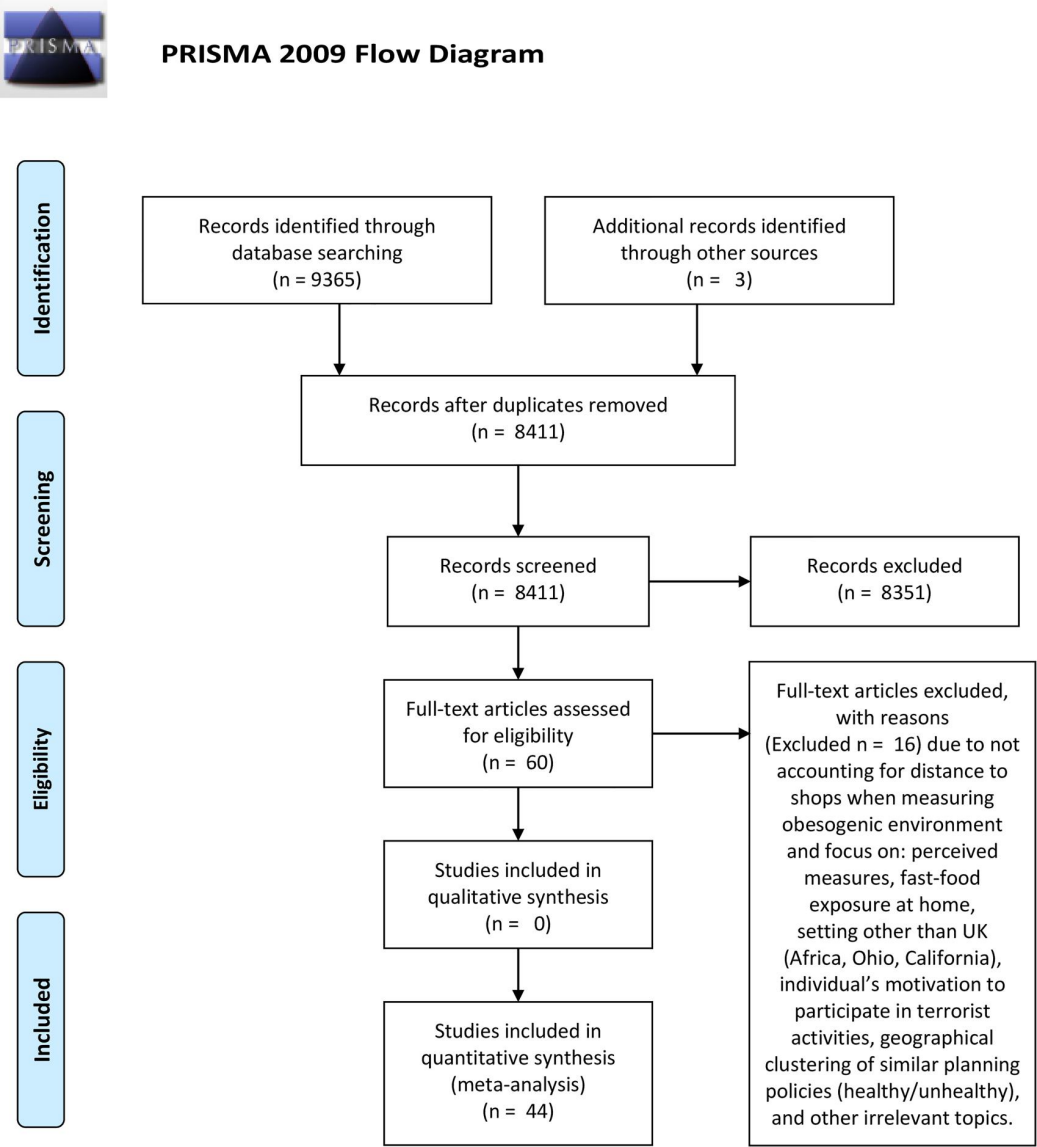
Preferred reporting items for systematic reviews and meta‐analyses flowchart (January 2010‐March 2021)

The search and screening were conducted according to the PRISMA 2009 protocol (Figure 1).^63^ The first author (ET) screened the search results against the identified eligibility criteria, and then extracted this information from each study. Due to the heterogeneity of the studies' methodologies, a meta‐analysis was not considered appropriate.

## 3 RESULTS

From the 9365 articles identified by the search strategy, 954 were removed due to duplication; an additional 3 articles were identified through other sources. As a result, 8411 articles were evaluated based on their title and abstract, leading to the removal of 8351. The remaining articles (*n* = 60) were downloaded for review against the inclusion and exclusion criteria, resulting in 44 unique studies included in the final sample (Figure 1).

There was very little between‐study similarity in methodology when modelling and measuring RFE, regarding the choice of dataset, definition of healthy versus unhealthy FROs or the choice of buffer cut‐offs. To investigate the impact of differences in choice of dataset and definition of neighborhood, Hobbs et al.^64^ compared two different datasets of food outlet locations and three different definitions of neighborhood, the latter including an 800‐m and a 2000‐m radial buffers around the geocoded home locations and neighborhood defined by identifying which Lower Layer Super Output Area (LSOA) an individual resided in, concluding that “other than for supermarkets, different definitions of neighborhood are broadly inconsequential in changing statistical inference.”^64^

Due to this heterogeneity in methodologies, as measures depend on many different factors, such as types of geography and data used, it is difficult to discuss them without providing this extra detail. Therefore, in addition to the methodology, measures in the context of a wider RFE, including neighborhood definition and food outlet type are discussed; for the classification of food access measures and detailed description of studies, see Figure S2 and Table S3.

### 3.1 General overview of the studies

Most studies exploring features of the RFE have been undertaken in local contexts, following a single case‐study approach. Twenty‐nine studies were carried out in England (two in London), five in Scotland, and one in Ireland ^65^; there were no studies carried out in Wales and nine studies were carried out in the United Kingdom. Associations were examined exclusively in both urban (*n* = 16) and rural settings (*n* = 4, from which three studies focused on Norfolk, being a rural county with 53% of its population designated as rural and only two primarily urban districts, and one study focused on Hampshire, with approximately 75% of Hampshire being classified as rural). Only two studies accounted for differences in urbanicity in England by including urban/rural status into the models as an independent variable^66^ or by utilizing geographically weighted regression (GWR) to draw trends for urban and rural areas,^67^ whereas one study^68^ explored food accessibility in four environmental settings (island, rural, small town, and urban) in Scotland. Finally, other studies explored RFE in different countries, including associations with lunchtime eating behaviors in youth across Canada, Scotland, and the United States,^69^ and building typology of neighborhoods based on environmental obesogenic characteristics of five European urban regions in Belgium, France, Hungary, the Netherlands, and the United Kingdom.^70^

All studies quantified access by average distance, with the exception of Smith et al.^68^ who used median travel time to grocery stores, including travel time to (a) the nearest store; (b) nearest store with fresh produce, including three categories of availability for 12 fresh produce items (1–4 items only, 5–8 items only, and 9–12 items only); and (c) nearest large store with fresh produce with a floor space in excess of 15,000 square feet. When considering outcome variable, the majority of studies examined association with adiposity (*n* = 22) and diet (*n* = 13), followed by deprivation (*n* = 3) and trends in foodscape (*n* = 3); single studies examined associations with socioeconomic differences in terms of educational attainment and household income,^71^ ethnic concentration in the neighborhood,^72^ and diabetes.^73^ Most often, association were examined among adults (*n* = 21, two studies specifically focused on pregnant women^45^, ^74^), followed by children (*n* = 11), teenagers (*n* = 5), elderly (*n* = 2), and infants (*n* = 1).

The studies present inconsistent and mixed findings (detailed summary is provided in Section 4) that might be due to (a) differences in theoretical and methodological constructs being used^75^ (including methods, definitions of “neighborhood,” exposure metrics and FROs types, and assessment of dietary intake); (b) focusing on exposures to FROs in residential neighborhoods^76^; and (c) dominance of cross‐sectional study design that may result in residual confounding.^77^ For the first time outside of North America, one study found a clear negative association of supermarket distance and density with diet in Ireland,^65^ which as suggested by authors, could be due to urban policy allowing small FROs to decline within urban areas due to competition from the development of large‐scale out‐of‐town shopping areas, showing that “Ireland is closer to the USA and Canada than it is to Europe and Australia in this regard.”^65^

#### 3.1.1 Data sources

In overall, the studies characterized RFE exposures purely on the basis of store location (two studies considered availability of healthy items “within” stores of interest^68^, ^78^), using both secondary and primary data sources, the latter including websites of major food retailers and symbol groups, and retailer's loyalty cards^79^; two studies used the fine‐detail, national scale of the environmental audit of built environment features,^80^, ^81^ and one study did not provide information about the outlet dataset used.^65^ In relation to health and diet outcome data, overall studies used survey‐based measures (one study used focus group discussions^82^), with the majority using self‐reported health and diet; several studies used objective measures taken by trained researchers, including food based audits,^80^ healthy food basket surveys,^78^ and anthropometric and body composition measures, the latter derived from the Fenland Study, the UK Biobank, and the English National Child Measurement Program.

#### 3.1.2 Food environment

The majority of studies focused on residential access (*n* = 31) followed by retail access (*n* = 8), with a growing acknowledgment of “activity spaces” (*n* = 2) and other relevant spatial contexts that are important in daily life, including schools (*n* = 4), work (*n* = 4) and routes (e.g., communing, *n* = 4), and some considerations for various modes of transport.^75^ Several studies examined more than one domain and/or examined access for two or more domains combined (e.g., residential and school^83^). When considering residential access, the focus was mainly on disadvantaged urban neighborhoods. When classifying FROs, researchers followed various systems, for example, as given by data providers (e.g., Newcastle City Council and Yell.com) to develop their own system^84^ or informed by the literature (e.g., as per a 22‐point classification system developed by Lake et al.^84^).^85^

Studies were classified on the basis of their focus on healthy versus unhealthy food stores, the latter including ready‐to‐eat food environments that sell food to be consumed instantly, following the standard supermarket/convenience division.^65^, ^78^ The major focus was on unhealthy outlets (*n* = 14 compared to *n* = 4 for exclusive focus on healthy, whereas *n* = 27 studies focused on both healthy and unhealthy outlets), within which FFs were most often studied, *n* = 33 (*n* = 8 for exclusive focus on FF); one study considered both permanent and mobile/nonpermanent locations, such as farmers' market stalls.^68^ Several studies examined the food environment in combination with other environmental features, such as physical environment features (*n* = 12) relating to walkability (e.g., residential density, street connectivity, land use mix), facilities for recreational PA, green spaces and air quality.

Molaodi et al.^72^ accounted for the widest range of PA facilities, both indoor (health fitness centers, ice rinks, indoor bowls, indoor tennis, sports halls, and swimming pools) and outdoor (athletics tracks, golf courses, ski slopes, synthetic turf pitches, and grass pitches). The study by Hobbs et al.^86^ is one of the first to consider both health‐promoting and ‐constraining neighborhood features. Burgoine et al.^66^ were the first to explore the influence of the environment defined as “walkability” on health and diet. Feuillet et al.^81^ considered the highest number of features (*n* = 56): walking and cycling related items, public transport, aesthetics, land use‐mix, grocery stores, FROs, and PA facilities. The study by Hawkesworth et al.^80^ is one of the largest to explore how the built environment may influence diet in older age, measuring diversity of food provision, local transport provision, and local food marketing. Recently, Daras et al.^87^ used 14 measures across three domains: retail environment (FF outlets, gambling outlets, pubs/bars/nightclubs, off‐licences, tobacconists), health services (General Practitioners [GPs], pharmacies, dentists, hospitals, leisure centers), and physical environment (green space and air quality) to create the physical environment index of “Access to Health Assets and Hazards” (AHAH).

### 3.2 Measures of food access

For describing the RFE, the majority of studies used network analysis in Geographic Information System (GIS) to estimate the shortest path between two locations on a road network and/or number of outlets within buffers. Studies tend to use either an address (*n* = 21) or a centroid (*n* = 18) to define food environment (10 studies used geographical centroid, three used population‐weighted centroid for Data Zones–DZs,^68^ postcode,^87^ and LSOAs^88^); single studies used unit postal code location in addition to geographic and population‐weighted centroid,^26^ and clusters of FF outlets defined as postcode areas in which there were three or more FF outlets.^45^ The majority of studies calculated distance using residential perspective (e.g., household, school), with the exception of two that used the catchment areas of stores.^78^, ^79^ Studies also tend to use person‐centered metrics (e.g., proximity, buffer), with the exception of eight studies that solely used definition of neighborhood as given by administratively defined areas, such as a LSOAs.

Slightly more studies used network distance (*n* = 15) rather than Euclidean distances (*n* = 11); 10 studies used a combination of the two and one study did not specify the distance definition.^82^ Within these categories, there was a lot of variance with respect to (a) approach (proximity, buffer, or Kernel); (b) buffer‐based metrics (counts, availability, density); (c) the very definition of density measure (absolute vs. relative); and (d) buffer definition.

The majority of studies measured locational access using a buffer approach only (*n* = 19), whereas 6 used proximity only and 12 used both approaches; 3 studies used Kernel density estimation.^26^, ^82^, ^89^ Within buffer metrics, most studies used densities (*n* = 23), including proportion (*n* = 6), followed by counts/availability (*n* = 18) and presence/absence of outlets (*n* = 5). Studies calculated densities of FROs per 10,000 and 1000 population, per square km, per capita, per mid‐2011 LSOA population estimates, per deprivation quintile and per concentration tertile, and per route length. More advanced relative densities included proportion of healthier food retailers relative to all outles,^70^, ^82^, ^90^ and less healthy retailers relative to (a) all outlets^71^, ^83^, ^91^; (b) FF combined with restaurants^91^; and (c) healthier outlets.^91^ Wilding et al.^89^ calculated relative exposure to unhealthy outlets by subtracting the density of healthy outlets from that of unhealthy outlets (positive values indicate a greater exposure to unhealthy outlets and vice versa).

For calculating buffers, a range of various cut‐offs were used, including 250 m, 400 m, 500 m, 800 m, 1 km/1 mile, 2 km, and different mixture of three or more cut‐offs; one study used 7% of the nearest observations at the address level,^82^ examining impact of adaptive bandwidth size on associations between food environment exposure and fruit and vegetable (F&V) intake. Various cut‐offs were found relevant for different settings (e.g., urban vs. rural or walking vs. driving); however, it remains unclear which representations, regardless of setting, best represent an actual usage of FROs.^32^, ^64^

Several studies used composite metrics and more complex measures, including sale of unhealthy foods as a percentage of total sales for the nine food categories (Unhealthy Foods Sales Percentage) for each store divided into quartiles,^79^ and total densities of all stores within 1‐km radius (an additive index comprising densities of all three ready‐to‐eat food outlet types).^73^ These novel measures require more detailed consideration and as such they are discussed separately in the follow up section. Our classification of measures including the food environment context is presented in Figure S2.

#### 3.2.1 Novel measures

Several studies used more complex measures that do not fit within definitions laid out earlier. They include (a) spatial access scores (combination of density and proximity) to healthy and less healthy FROs^70^; (b) ratio of spatial access scores to healthier and less healthy food retailers, being a relative indicator based on proximity, density, and variety^70^; (c) number of outlets along shortest distance route between home and school, divided by route length^6^; (d) diversity of RFE as given by spatial entropy score ranging from 0 to 1, with 0 representing a homogeneous area, covered by a single attribute, and 1 representing heterogeneity, where all attributes are equally distributed^80^; (e) a healthfulness score representing both type and number of FROs a person is exposed to within activity space, and a proxy of the healthfulness of the in‐store environment based on the availability of healthy and unhealthy foods in each outlet type^74^; and (f) accessibility score weighting the number of FROs in relation to their distance from an individual's residence^67^, ^92^; and weighted and averaged route exposures for people making multimodal journeys or using different modes of transport on different days.^75^, ^93^

#### 3.2.2 Variation between measures

An interesting group of studies examined variations between measures in terms of their effects on the studied associations, including correlation between subjective and objective measures,^94^ density and proximity measures,^26^, ^88^ availability and proximity measures,^95^ area‐ and person‐based measures,^71^ and absolute and relative measures.^71^ While some prior studies have compared how different access measure effect exposure estimates,^96^, ^97^ Thornton et al.^26^ were first in the United Kingdom to comprehensively explore a range of measures to assess impact on dietary outcomes, including proximity and density measures using different points‐of‐origin to vary levels of aggregation, all three approaches (Euclidean, road network buffers and density estimation) and buffer distances ranging from 0.4 to 5 km. Their results suggest there is an increased likelihood of conclusions with either Type‐I errors or Type‐II errors if research does not consider appropriateness of access measures; for example, greater access to supermarkets was associated with higher fruit and vegetable consumption for a number of measures used, but no association was apparent for counts of supermarkets within a 5 km road network buffer around an individual's unit postal code location, suggesting the potential risk of committing a Type‐II error.^26^

Burgoine et al.^88^ assessed differences between density and proximity metrics, and within types of density and types of proximity metrics, concluding that both metrics are largely comparable, and recommending moving toward a more standardized set of environmental metrics to ensure future comparability. Wilkins et al.^91^ employed different access metrics and definitions of outlet constructs to compare associations with weight status, concluding that both impacted observed associations, which may be due to different metrics capturing different dimensions of the RFE, and thus recommending using specific measures “in the context of the research question and the appropriate statistical frameworks and principles applied”^91^ ^(p. 10)^. Mason et al.^95^ compared FF outlet availability (number of stores within a 1‐km street‐network buffer around each participant's home address) categorized into three levels (0/1–2/3 or more) with an alternative measure of proximity to takeaway store, and found the latter yielded a weaker association among people living within 500 m of a store compared with those living further away. McDonald et al.^94^ investigated correspondence between individuals' subjective assessments of how well‐placed they are for everyday amenities, including food stores, and objective GIS‐modelled measures, concluding that individuals are more likely to perceive an amenity as closer or farther than it physically is. Finally, Maguire et al.^71^ compared area‐versus person‐centered measures and absolute versus relative measures when measuring socioeconomic variation in the foodscape, concluding that the association is sensitive to the metric used, and highlighting that studies may need to consider using multiple measures.

#### 3.2.3 Analytical method

The majority of studies used various regression methods (Poisson, logistic, multilevel, spatial) followed by sensitivity analyses; on average, studies used single methods. All methods adjusted for individual‐level covariates on the basis of priori literature, except five for which the use of confounders was inapplicable due to the study design. Studies also tend to adjust models for various local area characteristics, including area deprivation, urbanicity, residential density, PA facilities and food outlet types. Sarkar et al.^73^ considered novel exposures, including activity‐related variables, television screen time and metabolic equivalent of task‐h/week, and a nearby location of petrol‐filling stations or train or bus stations or terminals. Clary et al.^82^ adjusted for time spent at home, which may confound how the food environment relates to fruit and vegetable intake by computing a proxy for the “time spent in the neighborhood” variable.

Most studies have analyzed data at the global level accounting for the hierarchic nature of the data; two studies used GWR to estimate local models showing differences across space.^67^, ^82^ Statistical analyses were performed at various units of analysis; majority of studies used individual level of analysis and the highest level used was country.^69^ Data on FROs were also collapsed at various levels of geographies; several studies performed analysis on an individual level while collapsing data on FROs to a higher level of geography, for example, LSOA.^98^ One study measured environmental area characteristics at both LSOA and MSOA levels to test if the associations differ over a larger area of exposure.^89^ Most studies followed a cross‐sectional design and only four followed a longitudinal design, two of which assessed a causal link between food environment around schools and diet^76^ or BMI of students,^77^ whereas the third examined trends over time regarding links between area deprivation and the food environment,^99^ and the fourth averaged the densities for different types of outlets over several years (2008–2017) across LSOAs and MSOAs.^89^

## 4 DISCUSSION

The objective of this review was to classify the measures used to quantify the RFEs in the United Kingdom in recent years. Findings based on the review of 44 studies revealed that most of the existing work defined RFE exposure purely on the basis of store location. Second, measures were applied in the local context, focusing on relatively homogenous population size and exposure characteristics, and deprived urban areas. Third, when data were available at the national level, research has been undertaken for large geographical zones, which is not often useful for planning local policy interventions.^87^ As a result, other environmental determinants of nutrition behaviors (e.g., availability of healthy items “within” stores) and the impact of RFE on health in rural areas remain understudied; in addition, there was little focus on comparing urban and rural areas, with only one study of Scotland considering the full range of environmental settings, which included islands, rural areas, smaller towns, and urban centers,^68^ and the other two accounting for differences in the urbanicity status.^66^, ^67^ Finally, there are no studies using fine‐grained detail to analyze trends at the national level, making it suitable for understanding a spatial overview of food access indicators for various populations of interest (e.g., estimates of access for low‐income and postcode or by race and ethnicity), similar to the Food Access Research Atlas in the United States.^100^

There has been greater focus on less healthy FROs (*n* = 15 compared to *n* = 5 for focus on healthy FROs, *n* = 28 studies focused on both), FF in particular (*n* = 12 studies focused exclusively on FF outlets), and studies have often examined RFE in combination with other environmental features, allowing for a more realistic representation of contextual neighborhood factors and their contribution to diet and health. The former is in line with literature in the United States that has moved to describing FS, recognizing that obesogenic environments may be better explained by the impact of unhealthy RFE on dietary health as opposed shortages in healthy options. Most measures have considered geographic access alone and 15 studies considered other characteristics of areas or individual‐level mobility indicators, including: the neighborhood food marketing environment,^80^ recreational facilities,^6^, ^72^, ^81^, ^86^, ^95^, ^101^ walkability,^66^, ^80^, ^89^ land use mix and street connectivity,^6^ greenspaces,^87^, ^95^ air quality,^87^, ^89^ and various transport modes (walk, cycle, car, public transport).^26^, ^75^, ^80^, ^93^ In addition, Wilding et al.^89^ collated several environmental area characteristics, including greenspace (access to natural land), walkability, supermarket density, relative exposure to unhealthy food outlets, spaces for social interaction, particulate matter and nitrogen oxides, and one standout study explored agreement between people's perception of being well‐placed and objective presence of local amenities.^94^

Research has analyzed RFE characteristics for one time period, overlooking how food environments change over time; consequently, there is a considerable shortage of longitudinal studies allowing for drawing causal relations (*n* = 4), calling for future research to develop stronger causal models. Likewise, a spatial analysis of food access remains largely unaccounted (*n* = 4), similar to spatiotemporal considerations (*n* = 2), whereas US studies have already been using some advanced methods in this regard, such as Bayesian hierarchical modelling to analyze the spatiotemporal patterns of relative healthy food access (RHFA)^44^ that is calculated as the proportion of healthy FROs (healthy outlets/healthy plus unhealthy outlets) within 4 km from each small‐area, with the model measuring spatial autocorrelation, temporal trend, and spatiotemporal trends for small‐areas.

Systematic reviews of studies focused on RFEs in the United States^5^, ^34^ show a wide range of sophisticated metrics being in use for a long time, such as the gravity potential index, in which “facilities are weighted by their size and adjusted for the ‘friction of distance’.”^102^ Other research initiatives, such as consulting groups research and national‐level measures of access for guiding public policy,^36^ show similar characteristics; for example, Gallagher's^103^ Research & Consulting Group uses a Food Balance Score of an area while controlling for density (ratio between the distance to any grocer and the distance to any FF outlet using weighted average distance with a greater weight given to areas with larger number of residents), and United States Department of Agriculture uses measures that combine proximity with other tract‐level characteristics, such as the percentage of the population that both reside half‐mile from the nearest food outlet and have no access to a car. On the other hand, research in United Kingdom has tended to use simpler metrics, such as absolute densities, with the trend moving toward greater focus on relative indicators that is consistent with mounting evidence from other countries, including the United States, Australia and Canada, showing association between relative measures of exposure and various health outcomes^82^; only few recent studies used more complex methods, including Kernel estimations and composite metrics.

A growing number of studies have accounted for activity spaces and two or more domains combined (e.g., school and residential), recognizing that the geographical determinants of health are multidimensional in nature, therefore require univariate measures of accessibility,^104^ whereas reliance only on the characteristics of residential neighborhoods leads to underestimating the importance of exposure to foodscape^93^; others further argue that future research should consider the overall context in terms of varying types of neighborhoods, rather than examining environmental features in isolation.^81^ One example is the aforementioned AHAH index by Consumer Data and Research Centre (CDRC) that uses proximity to 11 outlets hazardous to health across three domains plus concentration of three air pollutants.^105^ An online map of AHAH domains and the overall index for the whole of the United Kingdom is available through an interactive web mapping tool. The program code to produce the AHAH index and components is open source and available through the GitHub repository. An updated version of AHAH (Version 2) includes additional fourth domain of air quality (nitrogen dioxide, particulate matter 10, sulfur dioxide). The index could be further developed by adding new relevant environmental features (e.g., access to sport facilities) and determining weighting.

When defining food access quantitatively, there are several outstanding measurement challenges. First, there is little agreement on how to measure “lack of healthy food” and what parameters to use. In addition to differences in food access definition, there are nearly as many measures as there are studies, leading to the conflicting evidence base and confusing policy messages; consequently, studies have called for a greater consistency across methods, including consideration of specific aspects of the methods.^71^ Second, area‐based measures, such as FD, require defining the area unit of analysis, that is, the neighborhoods or geographical areas on which to focus, leading to the Modified Areal Unit Problem. Third, there is a great methodological diversity regarding the choice of food outlet definitions (e.g., FF outlets were sometimes defined narrowly as chain outlets, or defined broadly to include cafes and Sandwich shops). To address the first two challenges, Thornton et al.^26^ have called for moving “from place‐based to people‐based measures of exposure,”^26^ allowing for understanding “true” environments. Others have called for rejecting a “growing misconception” that “the obesity epidemic can be subdued by addressing the built environment alone,”^66^ and recommend a multi‐faceted approach focusing on individual access, both direct and indirect, as given by individual‐level factors, such as age and gender, to understand association with energy intake.^66^

As the neighborhood definition that best represents actual food outlet usage remains unknown, several studies have advocated using various RFE measures, neighborhood definitions and measures of potential environmental confounders (e.g., street connectivity) to define and operationalize neighborhoods. Research also suggested that different measures may be capturing different dimensions of the RFE, such as accessibility, desire or normalization dimension, meaning only using multiple measures would adequately capture the RFE, as some measures may better capture a particular dimension than others, for example, the desire dimension could be better captured by the raw count of stores.^91^ Different metrics may also be more strongly/weakly correlated with factors confounding the relationship between RFE and obesity, leading to different statistical conclusions, however, further research is needed to confirm or reject this theory.^91^ Finally one study^95^ considered the fact that neighborhoods are multi‐dimensional, demonstrating possible interplay between multiple aspects of the built environment in the United Kingdom, and arguing that unhealthy RFE and greater density of green spaces in close proximity may undermine the potential benefits of formal PA facilities in terms of obesity risk.^95^ This further reinforces ongoing heterogeneity being problematic to policymakers but indeed may be necessary to fully understand complexities of RFE. Following an intuitive approach on convergence of results could perhaps provide a short‐term solution until better options become available; in other words, comparable measures that render similar results could be added to an arsenal of trustworthy measures for receiving greater consideration, whereas measures with contradicting results should be treated with caution requiring further examination.

Since defining food access is an evolving concept, RFE measures should be regularly improved and updated, followed by developing new more adequate measures.^36^ This, however, requires data at a small spatial scale that is not often readily available, as data may be costly, privately owned or incomplete at the national level.^87^ For example, in United Kingdom, health outcomes are available in a fashion through Hospital Episode Statistics (HES), managed by the National Health System (NHS), whereas general health is only really recorded in the Census and provided for the higher levels of geographies. CDRC recognizes these challenges, making its mission to utilize consumer data for academic research purposes. In the future, more effort should be made on the part of data holders to make data more easily accessible to researchers for speeding and strengthening the advance of human knowledge, and to create products and services that meet human needs and expand human capabilities; when possible, researchers should also make their data publicly available to advance research and promote open research community.

Results also suggest that research should use metrics relevant to the studied populations and effectively contribute to policy formulation.^91^ To guide policy, as well as to establish a common ground for an evidence base, a standardized set of features (e.g., buffer sizes) could be implemented for assessing how these features relate to obesity and other variables of interest, as suggested by Wilkins et al.,^91^ while considering interactions between them, that is specific relations between close environmental features compared with remote ones^81^; still, others argue that “due to the particularities of each research setting, reaching consensus on exposure measures may not be possible or even appropriate.”^70^ Since policy‐making requires instant evidence, converging on standards may not be possible in a situation when much is still unknown about what constitutes actual food outlet usage, however, being a pragmatic solution, this could be pursued by open research community in the future. Much like the international evidence, the UK evidence base is inconclusive and disparities in results necessitate further research, as evidence depends on the context and the choice of access measure and scale; while main results are discussed throughout this review, detailed summary of results for all studies, including implications and authors' recommendations is provided in Table S3.

Majority of studies found an evidence of positive association between variables of interest, including strong associations^101^, ^106^; however, the results were often not statistically significant; still, “the effect of individual foods combined could be important, particularly as even small differences in intake can impact on body weight over time.”^107^ Other studies found little or no consistent evidence for an association^69^, ^77^, ^80^, ^107^ or found a negative association.^45^ One study found a positive association for relative but not absolute measures, concluding that the latter may better capture the environmental risks for poor diet,^83^ whereas other study found the opposite, namely that GWR models using absolute measures outperformed models using relative measures^82^; others found that relative metrics give rise to larger effect sizes, further arguing that different relative metrics should not be interpreted synonymously,^91^ and some indication that absolute and relative measures of exposure may assess different aspects of the RFE.^70^

Hobbs et al.^64^ found little change in size and direction of associations across different definitions of neighborhood and datasets used; on the other hand, Wilkins et al.^91^ found that both the choice of outlet definition and the choice of RFE metric impacted associations under study. Burgoine et al.^88^ found that density and proximity metrics were largely comparable, with some exceptions, whereas Kraser et al.^98^ found the opposite, namely a positive relationship between the density of FF outlets per area and the obesity status of children, but no association between distance to the nearest FF outlet and child obesity. Finally, Wilding et al.^89^ longitudinally reaffirmed positive association between the relative density of unhealthy food outlets and BMI in children; in addition, when comparing associations at the two spatial scales (LSOA and MSOA), positive association was found for MSOA only, suggesting that the duration of exposure may bear a greater importance than the level of exposure; however, this needs to be further examined using data with greater temporal detail.^89^

The findings suggest that in urban settings the distribution of FROs may not be a major influence on diet and weight, possibly because most urban residents have reasonable food access^108^; on the other hand, results for Ireland show a clear impact of distance to larger FROs and density of outlets.^65^ The most accessible healthy areas were found to be concentrated in the periphery of the urban cores, whilst the least accessible healthy areas were located in the urban cores and the rural areas^87^; further, in majority of rural areas, increased accessibility of outlets was associated with FF consumption, whereas in some urban areas increased accessibility was associated with lack of consumption.^67^

This paper reports a systematic review of methods for assessing food accessibility and the evidence linking poor health outcome, including obesity and diet‐related environmental factors. Studies have tended to characterize RFE exposures purely on the basis of store location (as opposed to, e.g., availability of healthy items “within” stores) and to apply measures in the local context, limiting their overall generalizability. Simpler metrics, such as absolute densities, were used with the trend moving toward greater focus on relative indicators, less healthy FROs—FF in particular—and activity spaces. The association between RFE and diet and health in rural areas, as well as a spatiotemporal domain of food access, remains largely unaccounted. Evidence suggests that the duration of exposure may bear a greater importance than the level of exposure and that density‐based measures may better capture RFE when compared with proximity‐based measures, however, using more complex measures not necessarily produce better results. To move the field forward, studies have called for a greater focus on causality, individual access and the use of various measures, neighborhood definitions and potential confounders to capture different aspects and dimensions of the RFE, which requires using univariate measures of accessibility and considering the overall context in terms of varying types of neighborhoods.

Results of this review indicate that with no existing benchmark to compare and critically evaluate measures in terms of their strengths and weaknesses, ongoing heterogeneity could possibly be rendered by following an intuitive approach based on convergence of results. Overall, researchers should prioritize measures that may provide a more accurate and realistic account of people's lives, for example, in‐store food availability measures and activity spaces, and continue using individual socioeconomic characteristics and other relevant confounders (e.g., street connectivity). Upon confirming the dimensional theory of RFE, policy could be based on a multidimensional model providing a more nuanced assessment of the food environment, thus allowing for a better understanding of links between access and consumption. Since policy is an ongoing process, the aforementioned recommendations would constitute a short‐term solution to the problem of confusing evidence‐base; in the future, evidence could be gathered to converge on some useful standards and this process could be accelerated by greater data accessibility and open research community. Our recommendations are relevant to the United Kingdom, which the study addresses specifically, other developed countries and regions with similar data scarcity issues and socio‐economic trends, as well as European countries with underdeveloped economies.

## CONFLICT OF INTEREST

The authors declare no conflicts of interest.

## AUTHOR CONTRIBUTIONS

Elzbieta Titis: conceptualization (ideas; formulation of research goals and aims), data curation, formal analysis, methodology, writing the drafts. Rob Procter: writing, reviewing, and editing. Lukasz Walasek: writing, reviewing, editing, and revising.

## Supporting information

Supplementary Material 1

Supplementary Material 2

Supplementary Material 3
