# Supplementary material for: Assessing physical access to healthy food across United Kingdom: A systematic review of measures and findings
Source: Obes Sci Pract. 2021 Sep 15;8(2):233–46. doi: 10.1002/osp4.563 (PMC8976549; doi:10.1002/osp4.563)
Supplement: Supplementary file 1 — Supplementary Material 1 [file OSP4-8-233-s001.pdf]

SEARCH STRATEGY

Databases: Web of Science, PubMed, Scorpus

1. Scorpus

TITLE-ABS-KEY (food OR nutrition OR diet) **AND** TITLE-ABS-KEY (environment* OR communit* OR neighbo?rhood* OR "residence characteristics" OR grocer* OR supermarket* OR "food store*" OR "corner store*" OR "convenience store*" OR "food outlet*" OR "fast-food" OR restaurant* OR carryout OR takeaway OR "food environment" OR "food access" OR "food availability" OR "food desert*" OR "food savannah" OR "food oasis" OR "food swamp*") **AND** TITLE-ABS-KEY ("assess*" OR" measure*" OR "metric*" OR "instrument*") **AND**

TITLE-ABS-KEY ("United Kingdom" or UK or "U.K." or "Great Britain" or GB or "G.B." or England or Scotland or "Northern Ireland" or Wales) **AND**

TITLE-ABS-KEY (gb or "g.b." or britain* or (british* and not "british columbia") or uk or "u.k." or united kingdom* or (england* and not "new england") or northern ireland* or northern irish* or scotland* or scottish* or ((wales or "south wales") and not ("new south wales" or welsh*))) **AND**

TITLE-ABS-KEY ("North East" or "North West" or "Yorkshire and the Humber" or "West Midlands" or "East Midlands" or "South West" or "South East" or "East of England" or "Greater London") **AND**

TITLE-ABS-KEY (bath or "bath's" or (birmingham and not alabama*) or ("birmingham's" and not alabama*) or bradford or "bradford's" or brighton or "brighton's" or bristol or "bristol's" or carlisle or "carlisle's" or (cambridge and not (massachusetts* or boston* or harvard*)) or ("cambridge's" and not (massachusetts* or boston* or harvard*)) or (canterbury and not zealand*) or ("canterbury's" and not zealand*) or chelmsford or "chelmsford's" or chester or "chester's" or chichester or "chichester's" or coventry or "coventry's" or derby or "derby's" or (durham and not (carolina* or nc)) or ("durham's" and not (carolina* or nc)) or ely or "ely's" or exeter or "exeter's" or gloucester or "gloucester's" or hereford or "hereford's" or hull or "hull's" or lancaster or "lancaster's" or leeds* or leicester or "leicester's" or (lincoln and not nebraska*) or ("lincoln's" and not nebraska*) or (liverpool and not (new south wales* or nsw)) or ("liverpool's" and not (new south wales* or nsw)) or (london and not (ontario* or ont or toronto*)) or ("london's" and not (ontario or ont or toronto*)) or manchester or "manchester's" or (newcastle and not (new south wales* or nsw)) or ("newcastle's" and not (new south wales* or nsw)) or norwich or "norwich's" or nottingham or "nottingham's" or oxford or "oxford's" or peterborough or "peterborough's" or plymouth or "plymouth's" or portsmouth or "portsmouth's" or preston or "preston's" or ripon or "ripon's" or salford or "salford's" or salisbury or "salisbury's" or sheffield or "sheffield's" or southampton or "southampton's" or st albans or stoke or "stoke's" or sunderland or "sunderland's" or truro or "truro's" or wakefield or "wakefield's" or wells or westminster or "westminster's" or winchester or "winchester's" or wolverhampton or "wolverhampton's" or (worcester and not

(massachusetts* or boston* or harvard*)) or ("worcester's" and not (massachusetts* or boston* or harvard*)) or (york and not ("new york" or ny or ontario* or ont or toronto*)) or ("york's" and not ("new york" or ny or ontario* or ont or toronto*)) or bangor or "bangor's" or cardiff or "cardiff's" or newport or "newport's" or st asaph or "st asaph's" or st davids or swansea or "swansea's" or aberdeen or "aberdeen's" or dundee or "dundee's" or edinburgh or "edinburgh's" or glasgow or "glasgow's" or inverness or (perth and not australia*) or ("perth's" and not australia*) or stirling or "stirling's" or armagh or "armagh's" or belfast or "belfast's" or lisburn or "lisburn's" or londonderry or "londonderry's" or derry or "derry's" or newry or "newry's" **AND NOT**

TITLE-ABS-KEY (africa or americas or “north america” or “south america” or “antarctic regions” or “arctic regions” or asia or oceania or australia) **AND NOT**

TITLE-ABS-KEY (alabama or alaska or arizona or arkansas or aalifornia or aolorado or connecticut or delaware or florida or georgia or hawaii or idaho or illinois or indiana or iowa or kansas or kentucky or louisiana or maine or maryland or massachusetts or michigan or minnesota or mississippi or missouri or montana or nebraska or nevada or "new hampshire" or "new jersey" or "new mexico" or "new york" or "north carolina" or "north dakota" or ohio or oklahoma or oregon or pennsylvania or "rhode island" or "south carolina" or "south dakota" or tennessee or texas or utah or vermont or virginia or washington or "west virginia" or wisconsin or wyoming) **AND**

(LIMIT-TO (SRCTYPE,"j")) AND (LIMIT-TO (DOCTYPE,"ar")) AND (LIMIT-TO (LANGUAGE,"English")) **AND**

(LIMIT-TO (AFFILCOUNTRY,"United Kingdom"))

2. Web of science

(food OR nutrition OR diet) **and**

(environment* OR communit* OR neighbo?rhood* OR "residence characteristics" OR grocer* OR supermarket* OR "food store*" OR "corner store*" OR "convenience store*" OR "food outlet*" OR "fast-food" OR restaurant* OR carryout OR takeaway OR "food environment" OR "food access" OR "food availability" OR "food desert*" OR "food savannah" OR "food oasis" OR "food swamp*") **and**

("assess*" OR" measure*" OR "metric*" OR "instrument*") **and**

("United Kingdom" or UK or "U.K." or "Great Britain" or GB or "G.B." or England or Scotland or "Northern Ireland" or Wales or "North East" or "North West" or "Yorkshire and the Humber" or "West Midlands" or "East Midlands" or "South West" or "South East" or "East of England" or "Greater London" or gb or "g.b." or britain* or (british* not "british columbia") or uk or "u.k." or united kingdom* or (england* not "new england") or northern ireland* or

northern irish* or scotland* or scottish* or ((wales or "south wales") not ("new south wales" or welsh*)) or bath or "bath's" or (birmingham not alabama*) or ("birmingham's" not alabama*) or bradford or "bradford's" or brighton or "brighton's" or bristol or "bristol's" or carlisle or "carlisle's" or (cambridge not (massachusetts* or boston* or harvard*)) or ("cambridge's" not (massachusetts* or boston* or harvard*)) or (canterbury not zealand*) or ("canterbury's" not zealand*) or chelmsford or "chelmsford's" or chester or "chester's" or chichester or "chichester's" or coventry or "coventry's" or derby or "derby's" or (durham not (carolina* or nc)) or ("durham's" not (carolina* or nc)) or ely or "ely's" or exeter or "exeter's" or gloucester or "gloucester's" or hereford or "hereford's" or hull or "hull's" or lancaster or "lancaster's" or leeds* or leicester or "leicester's" or (lincoln not nebraska*) or ("lincoln's" not nebraska*) or (liverpool not (new south wales* or nsw)) or ("liverpool's" not (new south wales* or nsw)) or (london not (ontario* or ont or toronto*)) or ("london's" not (ontario or ont or toronto*)) or manchester or "manchester's" or (newcastle not (new south wales* or nsw)) or ("newcastle's" not (new south wales* or nsw)) or norwich or "norwich's" or nottingham or "nottingham's" or oxford or "oxford's" or peterborough or "peterborough's" or plymouth or "plymouth's" or portsmouth or "portsmouth's" or preston or "preston's" or ripon or "ripon's" or salford or "salford's" or salisbury or "salisbury's" or sheffield or "sheffield's" or southampton or "southampton's" or st albans or stoke or "stoke's" or sunderland or "sunderland's" or truro or "truro's" or wakefield or "wakefield's" or wells or westminster or "westminster's" or winchester or "winchester's" or wolverhampton or "wolverhampton's" or (worcester not (massachusetts* or boston* or harvard*)) or ("worcester's" (massachusetts* or boston* or harvard*)) or (york not ("new york" or ny or ontario* or ont or toronto*)) or ("york's" not ("new york" or ny or ontario* or ont or toronto*)) or bangor or "bangor's" or cardiff or "cardiff's" or newport or "newport's" or st asaph or "st asaph's" or st davids or swansea or "swansea's" or aberdeen or "aberdeen's" or dundee or "dundee's" or edinburgh or "edinburgh's" or glasgow or "glasgow's" or inverness or (perth not australia*) or ("perth's" and australia*) or stirling or "stirling's" or armagh or "armagh's" or belfast or "belfast's" or lisburn or "lisburn's" or londonderry or "londonderry's" or derry or "derry's" or newry or "newry's") **not**

(africa or americas or “north america” or “south america” or “antarctic regions” or “arctic regions” or asia or oceania or australia or alabama or alaska or arizona or arkansas or aalifornia or aolorado or connecticut or delaware or florida or georgia or hawaii or idaho or illinois or indiana or iowa or kansas or kentucky or louisiana or maine or maryland or massachusetts or michigan or minnesota or mississippi or missouri or montana or nebraska or nevada or "new hampshire" or "new jersey" or "new mexico" or "new york" or "north carolina" or "north dakota" or ohio or oklahoma or oregon or pennsylvania or "rhode island" or "south carolina" or "south dakota" or tennessee or texas or utah or vermont or virginia or washington or "west virginia" or wisconsin or wyoming)

3. Pubmed

food OR nutrition OR diet **and**

environment* OR communit* OR neighbo?rhood* OR "residence characteristics" OR grocer* OR supermarket* OR "food store*" OR "corner store*" OR "convenience store*" OR "food outlet*" OR "fast-food" OR restaurant* OR carryout OR takeaway OR "food environment" OR "food access" OR "food availability" OR "food desert*" OR "food savannah" OR "food oasis" OR "food swamp*" **and**

"assess*" OR" measure*" OR "metric*" OR "instrument*" **and**

"United Kingdom" or UK or "U.K." or Britain or "Great Britain" or GB or "G.B." or England or British or Scotland or Scottish or "Northern Ireland" or "Northern Irish" or Wales or "South Wales" or Welsh **or**

"North East" or "North West" or "Yorkshire and the Humber" or "West Midlands" or "East Midlands" or "South West" or "South East" or "East of England" or "Greater London" **not**

"new england" or "british columbia" or "new south wales" **or**

bath or "bath's" or ((birmingham or "birmingham's") not alabama) or bradford or "bradford's" or brighton or "brighton's" or bristol or "bristol's" or carlisle or "carlisle's" or (cambridge not (massachusetts or boston or harvard)) or ("cambridge's" not (massachusetts or boston or harvard)) or (canterbury not "new zealand") or ("canterbury's" not "new zealand") or chelmsford or "chelmsford's" or chester or "chester's" or chichester or "chichester's" or coventry or "coventry's" or derby or "derby's" or (durham not (carolina or nc)) or ("durham's" not (carolina or nc)) or ely or "ely's" or exeter or "exeter's" or gloucester or "gloucester's" or hereford or "hereford's" or hull or "hull's" or lancaster or "lancaster's" or leeds or leicester or "leicester's" or (lincoln not nebraska) or ("lincoln's" not nebraska) or (liverpool not ("new south wales" or nsw)) or ("liverpool's" not ("new south wales" or nsw)) or (london not (ontario or ont or toronto)) or ("london's" not (ontario or ont or toronto)) or manchester or "manchester's" or (newcastle not ("new south wales" or nsw)) or ("newcastle's" not ("new south wales" or nsw)) or norwich or "norwich's" or nottingham or "nottingham's" or oxford or "oxford's" or peterborough or "peterborough's" or plymouth or "plymouth's" or portsmouth or "portsmouth's" or preston or "preston's" or ripon or "ripon's" or salford or "salford's" or salisbury or "salisbury's" or sheffield or "sheffield's" or southampton or "southampton's" or st albans or stoke or "stoke's" or sunderland or "sunderland's" or truro or "truro's" or wakefield or "wakefield's" or wells or westminster or "westminster's" or winchester or "winchester's" or wolverhampton or "wolverhampton's" or (worcester not (massachusetts or boston or harvard)) or ("worcester's" not (massachusetts or boston or harvard)) or (york not ("new york" or ny or ontario or ont or toronto)) or ("york's" not ("new york" or ny or ontario or ont or toronto)) or bangor or "bangor's" or cardiff or "cardiff's" or newport or "newport's" or st asaph or "st asaph's" or st davids or swansea or "swansea's" or aberdeen or "aberdeen's" or dundee or "dundee's" or edinburgh or "edinburgh's" or glasgow or "glasgow's" or inverness or (perth not australia) or ("perth's" and australia) or stirling or

"stirling's" or armagh or "armagh's" or belfast or "belfast's" or lisburn or "lisburn's" or londonderry or "londonderry's" or derry or "derry's" or newry or "newry's" **and not**

Africa* or America* or Canad* or “north america” or “south america” or “antarctic regions” or “arctic regions” or Asia* or Oceani* or Australia* **or**

alabama or alaska or arizona or arkansas or aalifornia or aolorado or connecticut or delaware or florida or georgia or hawaii or idaho or illinois or indiana or iowa or kansas or kentucky or louisiana or maine or maryland or massachusetts or michigan or minnesota or mississippi or missouri or montana or nebraska or nevada or "new hampshire" or "new jersey" or "new mexico" or "new york" or "north carolina" or "north dakota" or ohio or oklahoma or oregon or pennsylvania or "rhode island" or "south carolina" or "south dakota" or tennessee or texas or utah or vermont or virginia or washington or "west virginia" or wisconsin or Wyoming **limit to**

(LIMIT-TO (SRCTYPE,"j")) AND (LIMIT-TO (DOCTYPE,"ar")) AND (LIMIT-TO (LANGUAGE,"English"))
