# Supplementary figures and images for: Assessing physical access to healthy food across United Kingdom: A systematic review of measures and findings
Source: Obes Sci Pract. 2021 Sep 15;8(2):233–46. doi: 10.1002/osp4.563 (PMC8976549; doi:10.1002/osp4.563)

FIGURE S2. Classification of measures

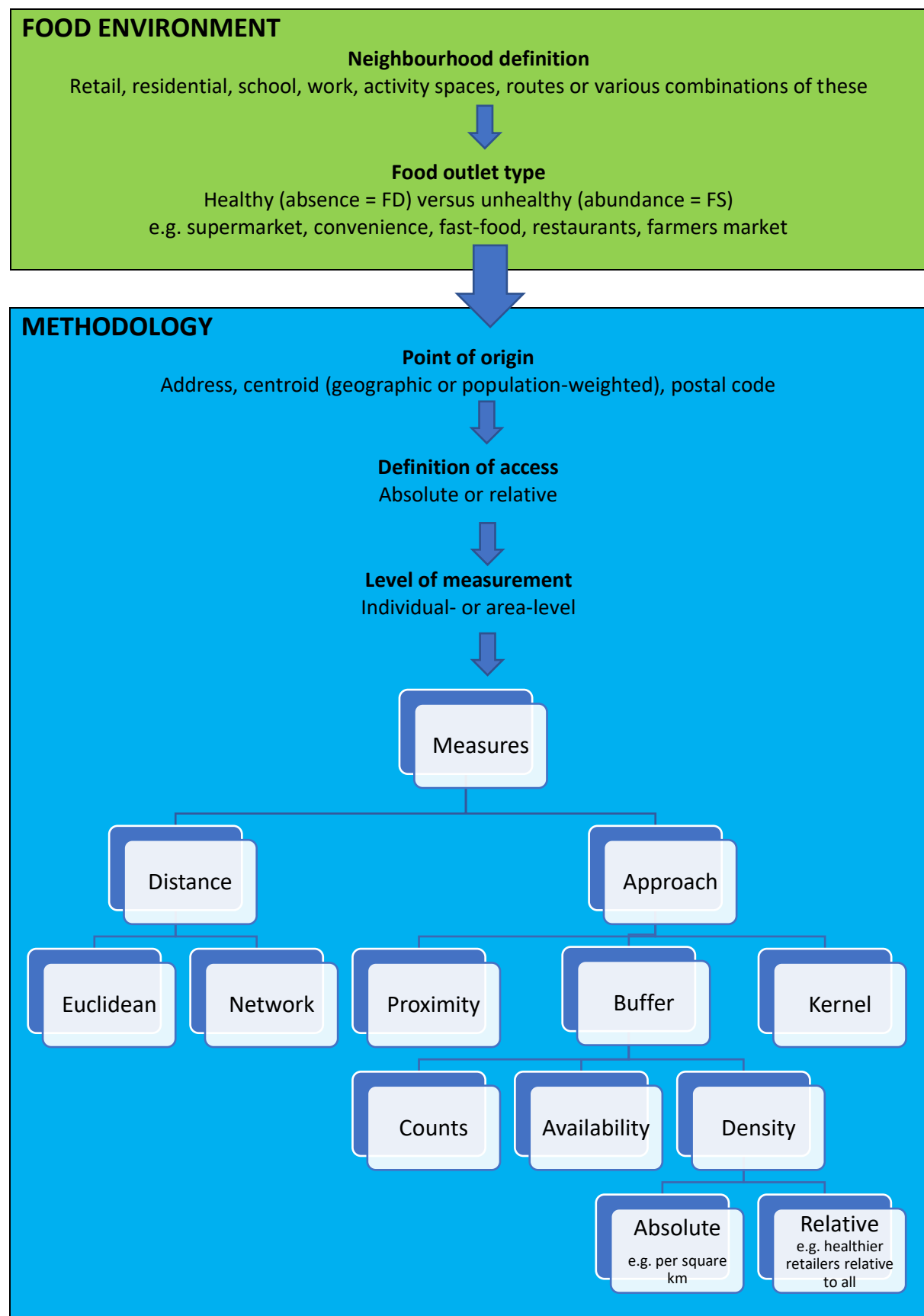

Supplement: Supplementary file 3 — Supplementary Material 3 [file OSP4-8-233-s003.pdf]
